# Supplementary material for: Comparison of null models for combination drug therapy reveals Hand model as biochemically most plausible
Source: Sci Rep. 2019 Feb 28;9:3002. doi: 10.1038/s41598-019-38907-x (PMC6395630; doi:10.1038/s41598-019-38907-x)
Supplement: Supplementary file 1 — Supplementary Information [file 41598_2019_38907_MOESM1_ESM.pdf]

Supplementary Information: Comparison of null models for combination drug therapy reveals Hand model as biochemically most plausible

Mark Sinzger[1,2], Jakob Vanhoefer[1,2], Carolin Loos[1,2], and Jan Hasenauer[1,2,3*]

[1]Helmholtz Zentrum München-German Research Center for Environmental Health, Institute of Computational Biology, Neuherberg 85764, Germany

[2]Chair of Mathematical Modeling of Biological Systems, Center for Mathematics, Technische Universität München, Garching 85748, Germany

[3]Faculty of Mathematics and Natural Sciences, University of Bonn, 53115 Bonn, Germany.

*jan.hasenauer@uni-bonn.de

December 13, 2018

1 The ODE governing the combined dose-effect curve of the Hand model

1.1 Derivation of the ODE

According to the Hand model, the dose-effect $f_{AB,\lambda}$ for the combined agent C_λ satisfies the ODE

$$f'_{AB,\lambda}(f^{-1}_{AB,\lambda}(x)) = \lambda \cdot f'_A(f_A^{-1}(x)) + (1-\lambda) \cdot f'_B(f_B^{-1}(x)). \tag{1}$$

This ODE for the combined dose-effect curve $f_{AB,\lambda}$ at fixed dose ratio λ can be derived using a Taylor expansion up to first order.

Denote by $x_1 = f_A(\,\mathrm{d}a + f_A^{-1}(x_0))$ and $x_2 = f_B(\,\mathrm{d}b + f_B^{-1}(x_1))$ the intermediate and final effect level after applying $\mathrm{d}a$ and $\mathrm{d}b$, respectively. Then the gains in effect due to A, B and their combination are

$$\begin{aligned} x_A &= x_1 - x_0 = f_A(\,\mathrm{d}a + f_A^{-1}(x_0)) - f_A(f_A^{-1}(x_0)) \\ x_B &= x_2 - x_1 = f_B(\,\mathrm{d}b + f_B^{-1}(x_1)) - f_B(f_B^{-1}(x_1)) \\ x_{AB} &= x_2 - x_0 = f_{AB,\lambda}(\,\mathrm{d}c + c) - f_{AB,\lambda}(c). \end{aligned}$$

By expanding we obtain

$$\begin{aligned} f_{AB,\lambda}(\,\mathrm{d}c + c) - f_{AB,\lambda}(c) =& (x_1 - x_0) + (x_2 - x_1) \\ =& \big(f_A(\,\mathrm{d}a + f_A^{-1}(x_0)) - f_A(f_A^{-1}(x_0))\big) + \big(f_B(\,\mathrm{d}b + f_B^{-1}(x_1)) - f_B(f_B^{-1}(x_1))\big) \\ =& \,\mathrm{d}a \cdot f'_A(f_A^{-1}(x_0)) + \,\mathrm{d}b \cdot f'_B(f_B^{-1}(x_1)) + o(\,\mathrm{d}c). \end{aligned}$$

Dividing by $\mathrm{d}c$ yields

$$\frac{f_{AB,\lambda}(c+\mathrm{d}c)-f_{AB,\lambda}(c)}{\mathrm{d}c} = \frac{\mathrm{d}a}{\mathrm{d}c} f_A'(f_A^{-1}(x_0)) + \frac{\mathrm{d}b}{\mathrm{d}c} f_B'(f_B^{-1}(x_1)) + o(1)$$
$$= \lambda f_A'(f_A^{-1}(x_0)) + (1-\lambda) f_B'(f_B^{-1}(x_1)) + o(1).$$

If we let tend $\mathrm{d}c \longrightarrow 0$, also $x_1 \to x_0$, the difference quotient approaches

$$\lim_{\mathrm{d}c\to 0} \frac{f_{AB,\lambda}(c+\mathrm{d}c)-f_{AB,\lambda}(c)}{\mathrm{d}c} = f_{AB,\lambda}'(c) = f_{AB,\lambda}'(f_{AB,\lambda}^{-1}(x_0))$$

and we get the above ODE.

1.2 Representations of the ODE

For completeness, and as they are referred to in the Supplementary Information, we mention here again the alternative representations of the ODE. This is an exact copy of the main article. Using the inverse derivative formula, we express the differential equation (1) as

$$\frac{1}{{f_{AB,\lambda}^{-1}}'(x)} = \frac{\lambda}{{f_A^{-1}}'(x)} + \frac{1-\lambda}{{f_B^{-1}}'(x)}, \tag{2}$$

or in integral representation

$$f_{AB,\lambda}^{-1}(x) = \int_0^x \left(\frac{\lambda}{{f_A^{-1}}'(y)} + \frac{1-\lambda}{{f_B^{-1}}'(y)} \right)^{-1} \mathrm{d}y. \tag{3}$$

In terms of sensitivities it is

$$s_{AB,\lambda}(x) = \lambda s_A(x) + (1-\lambda) s_B(x). \tag{4}$$

2 Effect-sensitivity curves

2.1 Derivation of the effect-sensitivity formula for Hill curves

The dose-effect behavior is often modeled by Hill curves. For Hill curves of the form

$$f_A(a) = E_{\min,A} + \frac{E_{\max,A}-E_{\min,A}}{1+\left(\frac{\mathrm{EC}_{50,A}}{a}\right)^{n_A}} = E_{\max,A} - \frac{(E_{\max,A}-E_{\min,A})\left(\frac{\mathrm{EC}_{50,A}}{a}\right)^{n_A}}{1+\left(\frac{\mathrm{EC}_{50,A}}{a}\right)^{n_A}},$$

the derivative is

$$f_A'(a) = \frac{n_A}{\mathrm{EC}_{50,A}\cdot(E_{\max,A}-E_{\min,A})} \left(f_A(a)-E_{\min,A}\right)^{1-\frac{1}{n_A}} \left(E_{\max,A}-f_A(a)\right)^{1+\frac{1}{n_A}}.$$

Thus, for the sensitivity we find

$$s_A(x) = f_A'(f_A^{-1}(x)) = \frac{n_A}{\mathrm{EC}_{50,A}\cdot(E_{\max,A}-E_{\min,A})} \left(x-E_{\min,A}\right)^{1-\frac{1}{n_A}} \left(E_{\max,A}-x\right)^{1+\frac{1}{n_A}}. \tag{5}$$

2.2 Conversion of dose-effect and effect-sensitivity curves

Starting with a strictly increasing, piecewise differentiable dose-effect function f_A, its inverse exists and its sensitivity is given by $s_A(x) = f_A'(f_A^{-1}(x))$. For a given positive piecewise continuous effect-sensitivity function s_A, the inverse f_A^{-1}

is obtained by

$$\begin{aligned} f_A^{-1}(x) &= f_A^{-1}(E_{\min,A}) + \int_{E_{\min,A}}^{x} {f_A^{-1}}'(y)\,\mathrm{d}y \\ &= f_A^{-1}(E_{\min,A}) + \int_{E_{\min,A}}^{x} \frac{1}{f_A'(f_A^{-1}(y))}\,\mathrm{d}y \\ &= f_A^{-1}(E_{\min,A}) + \int_{E_{\min,A}}^{x} \frac{1}{s_A(y)}\,\mathrm{d}y. \end{aligned}$$

Since by this calculation f_A^{-1} is a strictly increasing function, it is injective and its inverse f_A exists. Alternatively, f_A is obtained as the unique strictly increasing solution of the autonomous ODE $\frac{\mathrm{d}}{\mathrm{d}a}x(a) = s_A(x(a))$, $x(0) = E_{\min,A}$.

In order for the above calculation to be valid, we must assume that if $\lim_{x\to E_{\min,A}} s_A(x) = 0$, the integral

$$\int_{E_{\min,A}}^{E_{\min,A}+\varepsilon} \frac{1}{s_A(y)}\,\mathrm{d}y \tag{6}$$

is finite. For the class of s_A as given by the above formula for Hill curves, this assumption is satisfied, because $\frac{1}{s_A}$ behaves like $(x - E_{\min,A})^{-\gamma}$ near $E_{\min,A}$ with $\gamma = 1 - \frac{1}{n_A} \in (0,1)$ and thus integrates to a finite value. Note, that in the limit case $n_A = \infty, \mathrm{EC}_{50,A} = \infty$, such that $\frac{n}{\mathrm{EC}_{50,A}} = c_A$, s_A behaves like the logistic equation $s_A(x) = c_A\,(x - E_{\min,A})\,(E_{\max,A} - x)$ and the solution $x(a)$ of $\dot{x} = s_A(x)$ approaches $E_{\min,A}$ only in the limit $a \to -\infty$, not in a finite amount of dose.

Numerically the integral (6) or equivalently the ODE $\dot{x} = s_A(x), x(0) = E_{\min,A}$ must be treated with care whenever $s_A(E_{\min,A}) = 0$ because it allows the unfavored constant solution $x \equiv E_{\min,A}$ as well as solutions that are initially constant and exit $E_{\min,A}$ at an arbitrary dose value.

In the numerical implementation we solved this problem by setting the ODE's initial value to $x_0 = \varepsilon =$ `1e-7`. Details can be found in Sections 6 and 7.

3 Model properties

3.1 Proof of congruency of the Hand and the Loewe model assuming constant potency ratio

Let α be the constant potency ratio, i.e., for any effect level x, $f_A^{-1}(x) = \alpha f_B^{-1}(x)$, it holds for the derivatives:

$${f_A^{-1}}'(x) = \alpha {f_B^{-1}}'(x).$$

Then by (2) it follows for the combined curve $f_{AB,\lambda}$ of child agent C_λ

$$\frac{1}{{f_{AB,\lambda}^{-1}}'(x)} = (\lambda + \alpha(1-\lambda))\frac{1}{{f_A^{-1}}'(x)} \qquad \text{or equivalently} \qquad {f_A^{-1}}'(x) = (\lambda + \alpha(1-\lambda))\,{f_{AB,\lambda}^{-1}}'(x).$$

Provided that $f_A^{-1}(0) = 0 = f_{AB,\lambda}^{-1}(0)$, we get the relation

$$f_A^{-1} = (\lambda + \alpha(1-\lambda))\,f_{AB,\lambda}^{-1}. \tag{7}$$

Consequently $f_{AB,\lambda}, f_A$ and f_B are pairwise in constant potency relation. Let x be a given effect level. Now, we prove that the dose pair (a,b) lies on the straight line connecting $(f_A^{-1}(x), 0)$ and $(0, f_B^{-1}(x))$, if and only if $f_{AB,\lambda}(a+b) = x$

for $\lambda = \frac{a}{a+b}$:

$$\begin{aligned}
&\frac{a}{f_A^{-1}(x)} + \frac{b}{f_B^{-1}(x)} = 1 \\
\Leftrightarrow &(a+b)\left(\frac{a}{a+b} + \frac{\alpha b}{(a+b)}\right) = f_A^{-1}(x) \\
\Leftrightarrow &(a+b)\left(\lambda + \alpha(1-\lambda)\right) = f_A^{-1}(x) \\
\Leftrightarrow &(a+b) = f_{AB,\lambda}^{-1}(x).
\end{aligned}$$

3.2 Proof of the sham combination principle for the Hand, the Loewe and the Tallarida model

If $A = B$, then A and B have constant potency ratio $\alpha = 1$. In this case the Loewe, the Hand and the Tallarida models coincide, i.e.

$$E_L(a,b) = E_{T,A\to B}(a,b) = E_{T,B\to A}(a,b) = E_H(a,b) = f_A(a+b).$$

Then

$$f_{AA,\lambda}(c) = E(\lambda c, (1-\lambda)c) = f_A(c).$$

3.3 Disproof of the sham combination principle for the Bliss and the HSA model

The sham combination principle for the Bliss model is addressed in (Foucquier and Guedj, 2015). The HSA model predicts

$$f_{AA,\lambda}(c) = \max\{f_A(\lambda c), f_A((1-\lambda)c)\} < f_A(c)$$

if $\lambda \in (0,1)$ and f_A is strictly increasing.

3.4 Proof of the commutativity for the Hand, the Loewe, the Bliss and the HSA model

- The Hand model is commutative in A and B. $f_{AB,\lambda} = f_{BA,1-\lambda}$ because both satisfy the same ODE. Hence switching the roles of A and B along with their weights does not alter the combined dose-effect curve.
- The formulas for effects E_{Bliss} and E_{HSA} are symmetric in A and B.
- The Loewe isobole equation is symmetric in A and B. The isoboles determine the effect surface uniquely.

3.5 Disproof of the commutativity for the Tallarida model

The proof for the asymmetry in the Tallarida model is given in (Lorenzo and Sánchez-Marin, 2006).

3.6 Proof of the associative property for the Loewe and the Hand model

- The Loewe model: In terms of the combined curve $f_{AB,\lambda}$, we write the Loewe isobole equation as

$$\frac{\lambda f_{AB,\lambda}^{-1}(x)}{f_A^{-1}(x)} + \frac{(1-\lambda) f_{AB,\lambda}^{-1}(x)}{f_B^{-1}(x)} = 1 \qquad \text{or equivalently} \qquad f_{AB,\lambda}^{-1}(x) = \left(\frac{\lambda}{f_A^{-1}(x)} + \frac{(1-\lambda)}{f_B^{-1}(x)}\right)^{-1}.$$

Using analogous equations for $f_{AB,\mu}$ and $f_{C_\lambda C_\mu,\nu}$, we can calculate

$$\begin{aligned} f^{-1}_{C_\lambda C_\mu,\nu}(x) &= \left(\frac{\nu}{f^{-1}_{AB,\lambda}(x)} + \frac{(1-\nu)}{f^{-1}_{AB,\mu}(x)}\right)^{-1} \\ &= \left(\frac{\nu\lambda+(1-\nu)\mu}{f_A^{-1}(x)} + \frac{\nu(1-\lambda)+(1-\nu)(1-\mu)}{f_B^{-1}(x)}\right)^{-1} \\ &= f^{-1}_{AB,\nu\lambda+(1-\nu)\mu}(x), \end{aligned}$$

consequently $f_{C_\lambda C_\mu,\nu} = f_{AB,\nu\lambda+(1-\nu)\mu}$.

- The Hand model: The combined agents C_λ and C_μ satisfy (2)

$$\frac{1}{{f^{-1}_{C_\lambda}}'(x)} = \frac{\lambda}{{f_A^{-1}}'(x)} + \frac{1-\lambda}{{f_B^{-1}}'(x)}, \qquad \frac{1}{{f^{-1}_{C_\mu}}'(x)} = \frac{\mu}{{f_A^{-1}}'(x)} + \frac{1-\mu}{{f_B^{-1}}'(x)}.$$

For arbitrary $\nu \in [0,1]$, the grand child agent satisfies

$$\begin{aligned} \frac{1}{{f^{-1}_{C_\lambda C_\mu,\nu}}'(x)} &= \frac{\nu}{{f^{-1}_{C_\lambda}}'(x)} + \frac{1-\nu}{{f^{-1}_{C_\mu}}'(x)} \\ &= \frac{\nu\lambda+(1-\nu)\mu}{{f_A^{-1}}'(x)} + \frac{1-(\nu\lambda+(1-\nu)\mu)}{{f_B^{-1}}'(x)} \\ &= \frac{1}{{f^{-1}_{AB,\nu\lambda+(1-\nu)\mu}}'(x)} \end{aligned}$$

provided $f^{-1}_{C_\lambda C_\mu,\nu}(0) = 0 = f^{-1}_{AB,\nu\lambda+(1-\nu)\mu}(0)$ the grand child agent formed of the child agents C_λ and C_μ can indeed be formed by the parent agents A and B at appropriate ratio $\nu\lambda + (1-\nu)\mu$.

3.7 Disproof of the associativity property for the Tallarida, the Bliss and the HSA model

- The Tallarida model does not satisfy the associativity property. By the choice $\lambda = 0, \mu = 1$, the associativity property implies commutativity, which the Tallarida model violates.

- For the Bliss model, the case $\lambda = \mu = \nu = \frac{1}{2}, A = B$ shows that the associative property is violated because

$$2f_A(c) - f_A(c)^2 \neq f_A(2c) - \frac{1}{2}f_A(2c)^2.$$

- The HSA model is characterized by isoboles that form a rectangle with the dose axes. If the newly allocated coordinate axes are bent, the isobole will be reshaped to an angle of more than 90°. The characteristic property is then lost. Moreover, the isobole suggested by applying the HSA model on C_λ and C_μ encloses the original isobole, resulting in a smaller prediction value. This geometric interpretation of the associative property is displayed in the formal definition as well: From

$$f_{AB,\lambda}(c) = \max\{f_A(\lambda c), \quad f_B((1-\lambda)c)\}$$

we conclude that

$$
\begin{aligned}
f_{C_\lambda C_\mu,\nu}(c) &= \max\{f_{AB,\lambda}(\nu c), \quad f_{AB,\mu}((1-\nu)c)\} \\
&= \max\{f_A(\lambda\nu c), \quad f_B((1-\lambda)\nu c), \quad f_A((1-\nu)\mu c), \quad f_B((1-\nu)(1-\mu)c)\} \\
&< \max\{f_A((\nu\lambda + (1-\nu)\mu)c), \quad f_B((\nu(1-\lambda) + (1-\nu)(1-\mu))c)\} \\
&= f_{AB,\nu\lambda+(1-\nu)\mu}(c).
\end{aligned}
$$

4 Proof of the isoboles' convexity in the Hand model

The isoboles obtained from the Hand model are convex. They are strictly convex if and only if f_A and f_B exhibit a varying potency ratio.

We prove this property of the Hand model using (i) the integral representation (3) and (ii) the associativity property:

- Fix an effect level x, which is in the target domain of f_A and of f_B. Then for any $\lambda \in (0,1)$, it holds with $c = f_{AB,\lambda}^{-1}(x), a = \lambda c, b = (1-\lambda)c$, that
$$\frac{a}{f_A^{-1}(x)} + \frac{b}{f_B^{-1}(x)} \leq 1, \tag{8}$$
i.e., the pair (a,b) predicted by the Hand model to generate $E_H(a,b) = x$ lies below the Loewe straight isobole.
- To show convexity, apply the same argument with two child agents C_λ, C_μ instead of A, B and corresponding points
$$P_1 = (\lambda c_1, (1-\lambda)c_1), \quad P_2 = (\mu c_2, (1-\mu)c_2)$$
on the isobole at level x. By the associativity property, we can conclude that for any $\sigma \in [\lambda, \mu]$ the point $(\sigma c, (1-\sigma)c)$ on the Hand isobole at effect level x lies below the line segment through P_1 and P_2, completing the proof of the convexity property.

Now that the plan was established we carry out the steps.

- We proceed by first proving (8). For readability set $\tilde{A} = f_A^{-1}(x), \tilde{B} = f_B^{-1}(x)$. Factoring out c, and multiplying by $AB\lambda^{-1}(1-\lambda)^{-1}$, (8) is equivalent to
$$c\left(\frac{\tilde{A}}{\lambda} + \frac{\tilde{B}}{1-\lambda}\right) \leq \left(\frac{\tilde{A}}{\lambda}\right)\left(\frac{\tilde{B}}{1-\lambda}\right). \tag{9}$$
Integrating the rates $f_{AB,\lambda}^{-1'}, f_A^{-1'}, f_B^{-1'}$, we get the following representations
$$c = \int_0^x f_{AB,\lambda}^{-1'}(y)dy, \quad \frac{\tilde{A}}{\lambda} = \int_0^x \frac{f_A^{-1'}(y)}{\lambda}dy, \quad \frac{\tilde{B}}{1-\lambda} = \int_0^x \frac{f_B^{-1'}(y)}{1-\lambda}dy. \tag{10}$$
Define the second and third integrand as $h_A(y)$ and $h_B(y)$, respectively. Using the integral representation (3) of the ODE and the identity $(z^{-1}+w^{-1})^{-1} = \frac{zw}{z+w}$, which holds for any two positive real numbers:
$$c = \int_0^x \left(\frac{\lambda}{f_A^{-1'}(y)} + \frac{1-\lambda}{f_B^{-1'}(y)}\right)^{-1} \mathrm{d}y = \int_0^x \frac{h_A(y)h_B(y)}{h_A(y)+h_B(y)}\,\mathrm{d}y. \tag{11}$$
Substituting (10) and (11), (9) is equivalent to proving:
$$\int_0^x \frac{h_A(y)h_B(y)}{h_A(y)+h_B(y)}dy \cdot \int_0^x h_A(y) + h_B(y)dy \leq \int_0^x h_A(y)dy \int_0^x h_B(y)dy.$$

In order to establish this integral inequality, we use Cauchy-Schwarz

$$\langle f,g\rangle^2 - \|f\|^2 \|g\|^2 \leq 0 \tag{12}$$

for the scalar product

$$\langle f,g\rangle := \int_0^x f(y)g(y)dy$$

where we choose $f = \frac{h_A}{\sqrt{h_A+h_B}}, g = \sqrt{h_A+h_B}$. For readability we omit the integral bounds $0, x$ and the integration variable y when calculating

$$\begin{aligned}
\int \frac{h_A h_B}{h_A+h_B} \int (h_A+h_B) &= \int \frac{h_A((h_A+h_B)-h_A)}{h_A+h_B} \int (h_A+h_B) \\
&= \int h_A \left(\int h_A + \int h_B\right) - \int \frac{h_A^2}{h_A+h_B} \cdot \int (h_A+h_B) \\
&= \int h_A \int h_B + \left(\int h_A\right)^2 - \int \left(\frac{h_A}{\sqrt{h_A+h_B}}\right)^2 \int \left(\sqrt{h_A+h_B}\right)^2 \\
&\leq \int h_A \int h_B.
\end{aligned}$$

Furthermore, equality holds in (12) if and only if $f = \gamma g$ for some $\gamma \neq 0$. For the above f and g, this is equivalent to saying

$$(1-\gamma)h_A = \gamma h_B$$

or by plugging in the definitions of h_A, h_B

$$\frac{f_A^{-1'}}{f_B^{-1'}} = \frac{\lambda\gamma}{(1-\lambda)(1-\gamma)},$$

which means that f_A and f_B exhibit a constant potency ratio.

- It remains to prove that (8) indeed assures the convexity of the isobole. Let any three points $P_1 = (a_1, b_1), P_2 = (a_2, b_2)$ and $P_3 = (a_3, b_3)$ lie on the isobole. Rewrite $a_i = \lambda_i c_i, b_i = (1-\lambda_i)c_i$ for $\lambda_i = \frac{a_i}{a_i+b_i}$ and $c_i = \lambda_i^{-1} a_i$, upon relabeling we may assume $\lambda_1 < \lambda_3 < \lambda_2$. For convexity, it has to be proven that (a_3, b_3) lies inside the triangle $\Delta_1 = \Delta((0,0)^T, (a_1,b_1)^T, (a_2,b_2)^T)$. The linear transformation

$$T = (\lambda_1 - \lambda_2)^{-1} \begin{pmatrix} 1-\lambda_2 & -\lambda_2 \\ \lambda_1 - 1 & \lambda_1 \end{pmatrix}$$

converts the $([a],[b])$-plane into a $([c_1],[c_2])$-plane, with compound drugs

$$[c_i] = \lambda_i[a] + (1-\lambda_i)[b].$$

The assertion is then equivalent to $(a_3', b_3') := T(a_3, b_3)$ lying in $\Delta_2 = \Delta((0,0)^T, (c_1,0)^T, (0,c_2)^T) = T\Delta_1$. The dose pair (a_3', b_3') expresses the quantities of the drug compounds C_1, C_2 and has a ratio of

$$\nu = \frac{a_3'}{a_3'+b_3'} = \frac{a_3 - \lambda_2(a_3+b_3)}{(\lambda_1-\lambda_2)(a_3+b_3)} = \frac{\lambda_3 c_3 - \lambda_2 c_3}{(\lambda_1-\lambda_2)c_3} = \frac{\lambda_2-\lambda_3}{\lambda_2-\lambda_1}.$$

Using the associative property of the Hand model, we can apply the above derivation by replacing $f_A = f_{C_1}, f_B = f_{C_2}, f_{AB,\lambda} = f_{C_1C_2,\nu}$ and get

$$\frac{a_3'}{c_1} + \frac{b_3'}{c_2} \leq 1,$$

which establishes that (a_3', b_3') lies in Δ_2, and thus (a_3, b_3) lies in Δ_1. By this final argument we conclude convexity.

5 Derivation of the Loewe limit isobole

Let A and B be a partial and a full agent, i.e. $E_{\max,A} < E_{\max,B}$. Define $b^* := f_B^{-1}(E_{\max,A})$. Then the Loewe model assigns an effect value to all dose pairs (a, b) in the domain $D = \{(a, b)|b < b^*\}$ and $E_L(a, b) < E_{\max,A}$.

Proof: Take an arbitrary $(a_0, b_0) \in D$. Define the function $\varphi : [b_0, b^*] \to \mathbb{R}, \varphi(b) = f_A(a_b) - f_B(b)$, where a_b is the unique value such that $(a_b, 0), (a_0, b_0)$ and $(0, b)$ lie on one line. For b_0: $\varphi(b_0) = E_{\max,A} - f_B(b_0)$.

Then φ is a continuous function and $\varphi(b_0) > 0, \varphi(b^*) < 0$. Hence for some $b \in (b_0, b^*)$ we obtain

$$f_A(a_b) = f_B(b) =: x$$

so (a_0, b_0) lies on the isobole at effect level x. Since f_B is increasing $x = f_B(b) < f_B(b^*) = E_{\max,A}$.

On the other hand, all isoboles at effect levels $0 < x < E_{\max,A}$ lie in the domain D. The isoboles at effect levels $0 < x < E_{\max,A}$ cover the domain D. By continuity of E_L the limit isobole at the effect level $E_{\max,A}$ is forced to be the boundary of the set D, which is the horizontal line $\{(a, b)|b = b^*\}$.

6 Numerical implementation of the Hand model

The analysis of the experimental data from (O'Neil et al., 2016) was performed in MATLAB 2017a. The code can be found on GitHub: https://github.com/ICB-DCM/NullModels

For each cell line the coefficients of the Hill curve

$$f_A(a) = E_{\min,A} + \frac{E_{\max,A} - E_{\min,A}}{1 + \left(\frac{\mathrm{EC}_{50,A}}{a}\right)^{n_A}}$$

of the dose-effect curves of all drugs are fitted. We used multi-start optimization with starting values coming from a Latin hypercube and MATLAB's `lsqnonlin` for the least squares fit. The BIC was used to choose between a zero response-model and the Hill curve.

The different null models were implemented in MATLAB. The equation

$$\frac{a}{f_A^{-1}(x)} + \frac{b}{f_B^{-1}(x)} = 1, \tag{13}$$

which defines the reference effect for the Loewe model, was solved by bisection. If the dose pair (a, b) did not lie in the set $\{(a, b)|0 \le a < f_A^{-1}(E_{\max,B}), 0 \le b < f_B^{-1}(E_{\max,A})\}$, in which (13) is solvable, we extended the Loewe by $E_L(a, b) = \max\{f_A(a), f_B(b)\} = E_{\mathrm{HSA}}(a, b)$. This was done since the (axis parallel) HSA isobole can be seen as continuous extension of the Loewe isobole (see Section 5).

The combined dose-effect curve f_{AB} of the Hand model was computed by solving the ODE (4):

$$\frac{\mathrm{d}}{\mathrm{d}c} x(c) = s_{AB,\lambda}(x(c)) = \lambda s_A(x(c)) + (1 - \lambda) s_B(x(c))$$

The initial condition was set to $\varepsilon =$ `1e-7`, a value slightly above the ODE solvers absolute error tolerance. The MATLAB solver `ode15s` was used with the settings `AbsTol = 1e-8, RelTol = 1e-5` and the non-negative option.

7 Estimate on the Error

Numerically, the ODE (1) must not be solved starting in $x(0) = E_{\min,A}$, since the simulation would result in the unfavored solution which is constant $x \equiv E_{\min,A}$, unless the Hill coefficient is 1. If the ODE is simulated starting in $x(0) = E_{\min,A}+\varepsilon$, the error for the dose can be approximated. Assuming without loss of generality that $E_{\min,A} = 0$, we obtain

$$\begin{aligned} f_{AB,\lambda}^{-1}(\varepsilon) &= \int_0^\varepsilon \frac{1}{\lambda s_A(x) + (1-\lambda)s_B(x)} \mathrm{d}x \\ &\le \int_0^\varepsilon \frac{1}{\min\{s_A(x), s_B(x)\}} \mathrm{d}x \\ &\le \int_0^\varepsilon \frac{1}{s_A(x)} + \frac{1}{s_B(x)} \mathrm{d}x \\ &= f_A^{-1}(\varepsilon) + f_B^{-1}(\varepsilon). \end{aligned}$$

For $\lambda \in (.25, .75)$ we even get a better bound.

$$\begin{aligned} f_{AB,\lambda}^{-1}(\varepsilon) &= \int_0^\varepsilon \frac{1}{\lambda s_A(x) + (1-\lambda)s_B(x)} \mathrm{d}x \\ &= \int_0^\varepsilon \frac{\frac{1}{\lambda s_A(x)} \cdot \frac{1}{(1-\lambda)s_B}}{\frac{1}{\lambda s_A(x)} + \frac{1}{(1-\lambda)s_B(x)}} \mathrm{d}x \\ &\le \frac{1}{4} \left(\int_0^\varepsilon \frac{1}{\lambda s_A(x)} + \frac{1}{(1-\lambda)s_B(x)} \right) \mathrm{d}x \\ &= \frac{1}{4} \left(\frac{f_A^{-1}(\varepsilon)}{\lambda} + \frac{f_B^{-1}(\varepsilon)}{1-\lambda} \right) \\ &\le \frac{\min\{\lambda, 1-\lambda\}^{-1}}{4} \left(f_A^{-1}(\varepsilon) + f_B^{-1}(\varepsilon) \right), \end{aligned}$$

where we used the inequality of the geometric and arithmetic mean to estimate the integral.

In any case, the magnitude of the error $f_{AB,\lambda}^{-1}(\varepsilon)$ that results from starting the simulation at $x = \varepsilon$ is of the same order as $\max\{f_A^{-1}(\varepsilon), f_B^{-1}(\varepsilon)\}$. For a Hill curve f_A and ε small, we have

$$f_A^{-1}(\varepsilon) \approx \frac{\varepsilon^{\frac{1}{n}} \cdot \mathrm{EC}_{50}}{E_{\max,A}^{\frac{1}{n}}}.$$

For example, if $\varepsilon =$ `1e-7`, $E_{\max,A} =$ `1e0`, $n = 2, \mathrm{EC}_{50} =$`1e-1`, then $f_A^{-1}(\varepsilon) \approx 10^{-4.5}$.

References

Foucquier, J. and Guedj, M. (2015). Analysis of drug combinations: Current methodological landscape. *Pharmacology research & perspectives*, 3(3).

Lorenzo, J. I. and Sánchez-Marin, P. (2006). Comments on “Isobolographic analysis for combinations of a full and partial agonist: Curved isoboles”. *Journal of Pharmacology and Experimental Therapeutics*, 316(1):476–478.

O’Neil, J., Benita, Y., Feldman, I., Chenard, M., Roberts, B., Liu, Y., Li, J., Kral, A., Lejnine, S., Loboda, A., et al. (2016). An unbiased oncology compound screen to identify novel combination strategies. *Molecular cancer therapeutics*, 15(6):1155–1162.
